# Colorectal Cancer in Romania: Surgical Strategies, Survival, and Historical Trends in a 302-Patient Cohort

**DOI:** 10.3390/life15111686

**Published:** 2025-10-30

**Authors:** Laurențiu Augustus Barbu, Liliana Cercelaru, Valeriu Șurlin, Stelian-Stefaniță Mogoantă, Tiberiu Stefăniță Țenea Cojan, Nicolae-Dragoș Mărgăritescu, Ana-Maria Țenea Cojan, Valentina Căluianu, Gabriel Florin Răzvan Mogoș, Liviu Vasile

**Affiliations:** 1 Department of Surgery, Railway Clinical Hospital Craiova, University of Medicine and Pharmacy of Craiova, 2 Petru Rares Street, 200349 Craiova, Romania; laurentiu.barbu@umfcv.ro (L.A.B.); tiberiu.tenea@umfcv.ro (T.S.Ț.C.); valentina.andronache@yahoo.com (V.C.); gabriel.mogos@umfcv.ro (G.F.R.M.); 2 Department of Embryology and Anatomy, University of Medicine and Pharmacy of Craiova, 200349 Craiova, Romania; liliana.cercelaru@umfcv.ro; 3 Department of Surgery, Emergency County Hospital, University of Medicine and Pharmacy of Craiova, 2 Petru Rares Street, 200349 Craiova, Romania; vsurlin@gmail.com (V.Ș.); ssmogo@yahoo.com (S.-S.M.); vliviu777@yahoo.com (L.V.); 4 Faculty of Medicine, University of Medicine and Pharmacy of Craiova, 200349 Craiova, Romania; anamariatenea0324@gmail.com

**Keywords:** colorectal cancer, adenocarcinoma, Romania, survival, prognostic factors, surgical strategies, screening, oncology

## Abstract

**Background**: Colorectal cancer (CRC) is a leading cause of cancer-related mortality worldwide, with Romania reporting among the highest rates in the European Union. Regional outcome data remain scarce. **Methods**: We performed a retrospective cohort study of 302 patients with surgically treated colorectal adenocarcinoma at a Romanian tertiary hospital between 2003 and 2005, with a median follow-up of 60 months. Survival was analyzed using Kaplan–Meier and Cox regression. **Results**: Radical resection with R0 margins was achieved in 72% of cases. The overall 5-year survival was 38%, with significantly lower outcomes in advanced stages. Independent predictors of poor prognosis included advanced stage, emergency surgery, incomplete resection, and older age. **Conclusions**: Survival outcomes in this Romanian cohort were substantially lower than those reported in Western Europe, reflecting the burden of late-stage presentation. These findings emphasize the urgent need for nationwide CRC screening programs and wider access to modern multimodal therapies.

## 1. Introduction

Colorectal cancer (CRC) remains a major global health problem, ranking among the leading causes of cancer incidence and mortality. In 2020, more than 1.9 million new cases and over 930,000 deaths were recorded worldwide, with marked geographic variation in disease burden [1,2]. While the highest rates are traditionally reported in high-income countries, an upward trend is increasingly evident in low- and middle-income regions.

Romania reflects this global challenge, with CRC representing one of the most frequent malignancies and causes of cancer-related death. National and international statistics consistently show mortality rates above the European Union average, driven by late-stage diagnosis, limited screening coverage, and unequal access to multimodal therapy [3].

Despite these unfavorable trends, detailed regional data on treatment strategies and survival remain scarce. Unlike population-based registries or international estimates, our study provides granular, patient-level data from a Romanian tertiary center at a time when national screening and minimally invasive approaches were virtually absent. This cohort therefore offers both historical insights and a benchmark for evaluating the progress of colorectal cancer care in Romania, while also illustrating persistent survival gaps compared with Western countries. The present study aimed to describe surgical strategies, prognostic factors, and long-term outcomes in a consecutive cohort of CRC patients treated in a Romanian university center, thereby contributing region-specific evidence to the European and global literature.

## 2. Materials and Methods

### 2.1. Study Design and Setting

This retrospective observational cohort study included 302 consecutive patients diagnosed with colorectal adenocarcinoma and surgically treated in the Third Surgical Clinic of the Emergency County Clinical Hospital of Craiova, Romania, between 1 January 2003 and 31 December 2005. The hospital is a tertiary university center serving both urban and rural populations.

### 2.2. Inclusion and Exclusion Criteria

Eligible patients were adults (≥18 years) with histologically confirmed colorectal adenocarcinoma who underwent surgical treatment during the study period. Emergency interventions were included, provided that tumor resection was attempted. Patients undergoing only palliative derivations without resection were excluded. Additional exclusion criteria were incomplete clinical or follow-up data and histologies other than adenocarcinoma.

### 2.3. Data Collection

Clinical, surgical, pathological, and follow-up data were extracted from hospital records, surgical registries, and oncology charts. The following variables were collected:

**Demographics:** age, sex, and place of residence (urban vs. rural).

**Tumor characteristics:** location (colon vs. rectum; subsite), histological type, grade, TNM stage (AJCC/UICC 6th edition).

**Surgical data:** type of procedure (right/left hemicolectomy, anterior resection, abdominoperineal resection, subtotal colectomy, palliative procedures), surgical intent (curative vs. palliative), resection margin status.

**Treatment variables:** adjuvant or neoadjuvant radiotherapy/chemotherapy, timing and completion rates, emergency vs. elective surgery.

**Outcomes:** postoperative complications, length of hospital stay, reinterventions, and survival.

### 2.4. Definitions

For clarity, key definitions used in the analysis are summarized in Table 1.

### 2.5. Follow-Up

Patients were followed systematically in outpatient clinics, with scheduled clinical examinations, imaging, and laboratory tests. Additional survival data were verified using oncology records and national death registries. The median follow-up was 60 months (range, up to 84 months).

### 2.6. Statistical Analysis

Descriptive statistics were used to summarize baseline characteristics. Continuous variables were expressed as mean ± standard deviation (SD) or median with interquartile range (IQR). Categorical variables were reported as frequencies and percentages. Survival probabilities were estimated using Kaplan–Meier curves, with log-rank and Wilcoxon tests for group comparisons. Multivariate analysis was performed using Cox proportional hazards regression, including variables significant in univariate analysis (*p* < 0.05). Logistic, multinomial, and Poisson regression models were applied to identify predictors of surgical strategy, complications, and outcomes. Analyses were conducted using SPSS version 25.0 (IBM Corp., Armonk, NY, USA). Model assumptions were systematically verified. Proportional hazards were tested using Schoenfeld residuals and log-minus-log survival plots. Linearity of continuous variables was assessed through Martingale residual plots, while multicollinearity was evaluated using variance inflation factors (VIF), all below 2. For logistic and Poisson models, goodness of fit was examined using Hosmer–Lemeshow and deviance statistics. No major violations of model assumptions were detected.

### 2.7. Ethics Statement

The study was conducted in accordance with the Declaration of Helsinki and was approved by the Ethics Committee of the Emergency County Clinical Hospital of Craiova (**approval number** 44473/19 September 2025). Written informed consent for the use of anonymized medical data was obtained from all patients at admission.

## 3. Results

Baseline characteristics of the cohort are presented in Table 2. The mean age was 65 years, with most patients aged 60–79 years, and males were predominant. The majority of patients resided in urban areas. Tumors were more often located in the colon than in the rectum, and nearly 60% were diagnosed at an advanced stage (III–IV).

**Figure 1:**
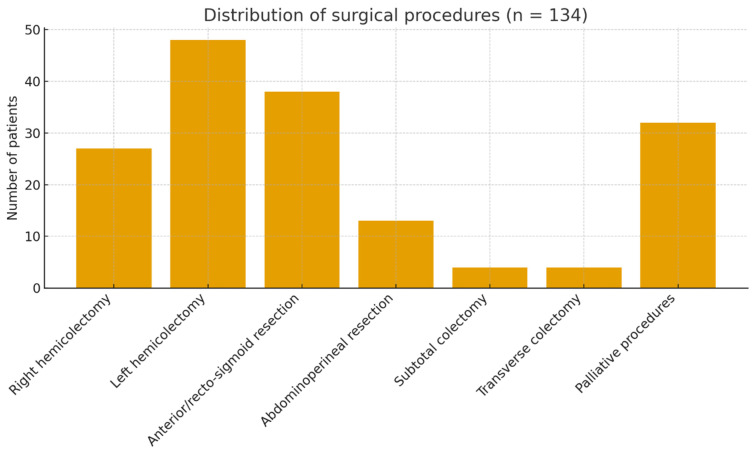
**Distribution of surgical procedures in operated patients (*n* = 134).** Bar chart showing the frequency of surgical strategies. Radical resections (right/left hemicolectomy, anterior/recto-sigmoid, abdominoperineal, subtotal/transverse colectomy) predominated, while palliative procedures represented 24% of cases.

Of the 302 patients included in the cohort, 134 underwent surgical resection with curative intent and were analyzed for surgical strategy distribution (Figure 1). The remaining patients either underwent palliative derivation procedures or were deemed inoperable due to advanced disease or comorbidities and were therefore not included in this specific analysis. All 302 patients were, however, included in the overall survival analysis.

Survival outcomes are presented in Table 3. Patients diagnosed in early stages and those undergoing R0 resections had markedly better survival rates compared to advanced-stage patients and those with residual disease.

**Figure 2:**
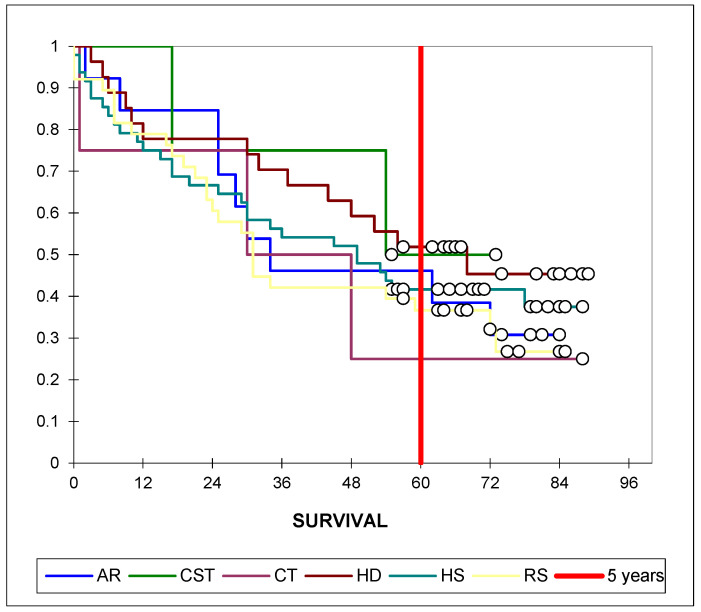
**Kaplan–Meier overall survival by tumor location.** Kaplan–Meier survival curves by tumor location (AR = ascending rectum, CST = cecum/ascending colon, CT = transverse colon, HD = descending colon, HS = sigmoid colon, RS = rectosigmoid junction). The red vertical line marks 60 months. Median survival with 95% CIs is shown in Table 3. No significant differences were observed between groups (log-rank *p* = 0.744).

Overall survival was evaluated by tumor location using Kaplan–Meier analysis. Survival curves showed only minor differences across colonic subsites, with no statistically significant variation in 5-year survival probabilities (log-rank *p* = 0.744) (Figure 2).

Chi-square tests revealed no significant associations between tumor location and surgical setting, histological grade, stage, complications, or macroscopic appearance (all *p* > 0.05) (Table 4). A significant association was observed between tumor location and survival duration (χ^2^ = 28.697, *p* = 0.026), but this was no longer significant when survival was dichotomized as <5 years versus ≥5 years (*p* = 0.590). Notably, Kaplan–Meier analysis showed no significant difference in overall survival between tumor locations (log-rank *p* = 0.744), indicating that the Chi-square result likely reflects distributional differences rather than a true prognostic effect.

Binary logistic regression (Table 5) showed that advanced stage (III–IV), emergency presentation, and incomplete resection (R1–R2) were strong independent predictors of reduced 5-year survival (all *p* < 0.01). Older age (>70 years) was also associated with poorer prognosis (*p* = 0.025). In contrast, sex, residence, and tumor location had no significant prognostic impact (all *p* > 0.05).

Multivariate Cox regression (Table 6) confirmed advanced stage (III–IV), emergency presentation, and incomplete resection (R1–R2) as independent predictors of reduced overall survival (all *p* < 0.01). Older age (>70 years) was also linked to higher mortality (*p* = 0.020). Sex, residence, and tumor location showed no significant prognostic impact (all *p* > 0.05).

Ordinal logistic regression (Table 7) showed that older age (>70 years), rural residence, and emergency presentation were significantly associated with advanced stage at diagnosis (all *p* < 0.05). Sex and tumor location were not significantly related to stage distribution (*p* > 0.05).

Multinomial logistic regression (Table 8) identified tumor location as the main determinant of surgical strategy: right-sided tumors predicted right hemicolectomy, while rectal tumors predicted abdominoperineal resection (both *p* < 0.001). Advanced stage increased the likelihood of palliative surgery (*p* = 0.002), and complicated tumors were associated with emergency presentation (*p* = 0.004). Older age (>70 years) showed only a non-significant trend toward palliation (*p* = 0.090).

Poisson regression (Table 9) showed that older age (>70 years), advanced stage (III–IV), and emergency surgery were significantly associated with higher rates of postoperative complications (all *p* < 0.05). Sex, resection margins, and tumor location were not significantly related to complication risk (all *p* > 0.05).

Negative binomial regression (Table 10) showed that older age (>70 years), advanced stage (III–IV), emergency presentation, and postoperative complications were significant predictors of prolonged hospitalization (all *p* < 0.05). Postoperative complications had the strongest impact on length of stay, while sex and resection margins were not significant (*p* > 0.05).

Logistic regression (Table 11) showed that advanced stage (III–IV) and emergency presentation significantly reduced the likelihood of achieving complete (R0) resection (*p* < 0.01). Rectal location and older age (>70 years) were not significantly associated with resection completeness (*p* > 0.05).

## 4. Discussion

### 4.1. Epidemiology and Burden of Disease

Our study (2003–2005 cohort) revealed a relatively low incidence (7–8 per 100,000) but with a high mortality-to-incidence ratio (≈0.67), suggesting late diagnosis and limited access to modern therapies. Mortality ranged between 5 and 5.5 per 100,000, with male predominance (M/F ratio = 1.43:1) and a mean age at diagnosis of 65 years.

Recent data highlight a dramatic increase in the burden of disease in Romania. According to the OECD/European Commission 2025 country profile, age-standardized colorectal cancer mortality in 2021 was 49 per 100,000 in men and 23 per 100,000 in women, which are 35% and 13% higher than the EU average, respectively [4,5,6]. In contrast, EU trends show a steady decline (2024 predictions: –4.8% in men and –9.5% in women compared with 2018) [5], while in the USA the mortality-to-incidence ratio is approximately 0.35, reflecting the effectiveness of screening and innovative therapies [5].

A direct comparison shows a four- to five-fold increase in national rates over the last two decades, a persistent late-stage diagnosis, and a progressive divergence from the declining trends observed in the EU and USA. These findings reflect the absence of an effective nationwide colorectal cancer screening program in Romania and limited access to innovative treatment modalities [1,3,5].

When comparing our historical cohort (2003–2005) with contemporary data from Romania, the EU, and the USA, a striking increase in incidence and persistently high mortality can be observed. Despite alignment in sex ratio and mean age at diagnosis, Romania continues to display a mortality-to-incidence ratio of ~0.70, in contrast to ~0.35–0.40 in Western countries, highlighting gaps in early detection and treatment availability (Table 12).

### 4.2. Strengths and Limitations

This study has several notable strengths. A major advantage is the combined retrospective and prospective design, which allowed us to capture both historical patterns and real-time clinical trends. In addition, the cohort was comprehensively followed up, ensuring complete outcome data and minimizing attrition bias. These methodological features enhance the reliability of our results and provide a robust basis for international comparison.

Nevertheless, several limitations must be acknowledged. The study was conducted in a single tertiary center, which may limit the generalizability of the findings and does not fully reflect the heterogeneity of colorectal cancer care across Romania. Our center had relatively better access to surgical expertise, diagnostic procedures, and oncology services compared with smaller regional hospitals. In other areas, limited infrastructure and delayed access to care may result in more advanced disease at diagnosis and poorer outcomes. Consequently, the generalizability of our findings to the wider Romanian population is restricted, and future multicenter studies are needed to provide a more comprehensive national picture. Although the sample size was fully followed up, it remains relatively limited compared with large multicenter registries.

Furthermore, staging and treatment protocols applied during the study period (2003–2005) differ substantially from current standards, limiting direct comparability with recent cohorts. Another important limitation is the absence of standardized reporting of surgical quality indicators, such as circumferential resection margin (CRM) status and total lymph node count, which reflects the limited oncological standardization of the period. Moreover, inclusion and exclusion criteria were less granular than modern trial standards, which should be taken into account when interpreting these results.

Another limitation is the lack of standardized and detailed information on chemotherapy regimens, which were largely limited to 5-FU–based protocols, as well as the absence of molecular or genetic profiling. These factors restricted biological stratification and prevented the inclusion of treatment variations in the multivariate analysis. These therapeutic constraints may have contributed to the poor overall survival observed in advanced-stage patients.

Given the retrospective nature of data collection, several measures were implemented to ensure accuracy and completeness. Data were extracted from multiple independent sources, including surgical logs, hospital archives, oncology registries, and death records, and cross-checked by two independent investigators. Missing or inconsistent entries were verified against original patient files whenever possible. Nevertheless, potential sources of bias remain, including incomplete documentation, differences in reporting standards over time, and variability in data recording practices. Although model assumptions were systematically assessed, the possibility of residual confounding or unrecognized violations (e.g., non-proportional hazards or collinearity) cannot be entirely excluded. These factors may have introduced misclassification or selection bias, potentially underestimating or diluting some associations observed in the analysis.

These limitations underscore the need for multicenter prospective studies, standardized surgical and pathological reporting, and the integration of molecular profiling to better align Romanian colorectal cancer cohorts with international standards.

### 4.3. Future Directions

Future research in Romania should expand beyond single-center studies to multicenter and nationwide cohorts or registries, ensuring better representativeness and real-time monitoring of colorectal cancer (CRC) incidence, treatment patterns, and outcomes. Such initiatives would allow benchmarking against European and international standards and provide the evidence base required for health policy planning. The systematic evaluation of pilot CRC screening programs launched after 2019 should also be prioritized, with particular attention to participation rates, early-stage detection, and survival benefits, as these data will be essential for the nationwide implementation of effective screening.

In parallel, integrating epidemiological, clinical, and molecular data into national databases is crucial for advancing precision oncology. Incorporating biomarker testing (e.g., KRAS, NRAS, BRAF, MSI) into routine practice would align Romanian oncology with international standards and support personalized treatment strategies. Finally, long-term prospective studies that combine clinical, molecular, and public health perspectives will be critical for monitoring trends, assessing preventive interventions, and guiding national cancer control policies.

### 4.4. Therapeutic Strategies

#### 4.4.1. Types of Surgical Procedures and Intent

Between 2003 and 2005, a total of 134 colorectal resections with curative intent were performed. The distribution was as follows: right hemicolectomy (27 cases, 20.1%), transverse colectomy (4 cases, 3.0%), left hemicolectomy (48 cases, 35.8%), rectosigmoid resection (Dixon procedure, 38 cases, 28.4%), abdominoperineal resection (13 cases, 9.7%), and subtotal colectomy (4 cases, 3.0%). Palliative procedures, including derivations without resection and Hartmann’s reversals, were excluded from this analysis. Notably, no laparoscopic resections were performed during this time, reflecting the limited implementation of minimally invasive approaches in Romania in the early 2000s. Laparoscopic and robotic surgery have been consistently associated with reduced postoperative morbidity, shorter length of stay, and faster recovery, without compromising oncological outcomes, as demonstrated in multiple randomized trials (COST, CLASICC, COLOR). The exclusive use of open procedures in our series may therefore have contributed to the higher complication rates, prolonged hospitalization, and slower postoperative recovery compared with modern standards. This limitation reflects the surgical landscape in Romania during the early 2000s and partly explains the gap between our historical outcomes and contemporary international benchmarks. Median survival varied across procedures (e.g., 57 months for right hemicolectomy, 47.6 months for left hemicolectomy, and 41.2 months for rectosigmoid resection), although differences were not statistically significant at log-rank/Wilcoxon testing. R-status (R0 vs. R1/R2) and circumferential resection margin (CRM, rectal cancer) were not systematically reported in this cohort, limiting direct comparison with international quality indicators.

Surgical strategies reflected both curative intent and the burden of advanced disease. Radical resections were feasible in approximately three-quarters of patients, with R0 margins achieved in 72% of cases. However, the remaining quarter of patients underwent palliative procedures, and a significant proportion of resections were incomplete (R1–R2), underscoring the challenge of offering curative surgery in advanced presentations.

The burden of complications and longer hospitalization times was closely linked to age, stage, and emergency interventions. These findings point to the need for better preoperative optimization and careful surgical planning to mitigate risks in vulnerable subgroups [8].

#### 4.4.2. Comparison with International Guidelines

For localized colon cancer, NCCN and ESMO guidelines recommend segmental oncologic resection with high vascular ligation and regional lymphadenectomy, examining at least 12 nodes to secure an R0 resection [9,10]. Adjuvant chemotherapy with FOLFOX or CAPOX is standard in stage III and in stage II with high-risk features such as T4 tumors, lymphovascular invasion, or <12 nodes retrieved [11,12].

In rectal cancer, the gold standard remains total mesorectal excision (TME) with negative circumferential margins. Current guidelines endorse total neoadjuvant therapy (TNT), combining chemotherapy and (chemo)radiotherapy, followed by TME or, in selected complete responders, a watch-and-wait strategy [13,14]. For early lesions or malignant polyps, ESGE 2024 recommends endoscopic approaches (cold snare polypectomy, EMR, or ESD) when feasible, with histologic assessment of invasion depth to guide surgical decisions [15].

Minimally invasive surgery has since become standard in many centers, supported by randomized trials (COST, CLASICC, COLOR) confirming the oncological safety of laparoscopic colectomy [16,17,18]. In contrast, none of the resections in our cohort were laparoscopic, reflecting the limited uptake of minimally invasive techniques in Romania during 2003–2005.

#### 4.4.3. Access to Adjuvant and Neoadjuvant Therapies in the Romanian Context (2003–2005)

Of the 134 patients, 103 (76.9%) received adjuvant and/or neoadjuvant radio/chemotherapy. Among rectal cancer cases, 23 patients with middle or inferior rectal tumors underwent preoperative radiotherapy, while 42 patients (31.3%) received postoperative radiotherapy. Only half (23/46) of those eligible for neoadjuvant therapy completed the full regimen within the recommended 30–45 days before surgery. Adjuvant chemotherapy was predominantly 5-FU ± leucovorin, with limited access to oxaliplatin and gradual introduction of FOLFOX/FOLFIRI regimens. Data regarding the management of disease recurrence were not systematically collected in this historical cohort. At the time (2003–2005), access to second-line systemic therapy in Romania was limited, and most patients received either best supportive care or 5-FU rechallenge, with very restricted availability of oxaliplatin- or irinotecan-based regimens. Targeted therapies and immunotherapy were not accessible. This therapeutic context likely contributed to the poor overall survival observed in advanced-stage patients.

In Romania during 2003–2005, minimally invasive approaches such as laparoscopy or robotics were not available, compliance with neoadjuvant regimens was suboptimal, and systemic therapy relied heavily on fluoropyrimidines. These limitations, together with incomplete adoption of multimodal protocols and inconsistent reporting of resection margin status, likely contributed to poorer outcomes compared with Western European and North American cohorts. By contrast, current NCCN and ESMO guidelines recommend FOLFOX or CAPOX for stage III colon cancer (and selected high-risk stage II), total neoadjuvant therapy (TNT) for locally advanced rectal cancer, and routine molecular profiling (RAS, BRAF, MSI-H/dMMR) to guide targeted and immunotherapies in metastatic disease [11,13,14,19,20,21].

### 4.5. Prognostic Factors

The demographic profile of our cohort, with a mean age of 65 years and a male-to-female ratio of 1.43:1, is consistent with international reports on colorectal cancer [22,23]. More than 60% of cases were diagnosed in advanced stages (III–IV), reflecting the absence of nationwide screening and limited access to early endoscopy in Romania [3].

Survival analysis confirmed the prognostic impact of stage. The overall 5-year survival of 38% was substantially lower than the 55–60% reported in Western registries [24,25]. Patients with stage I–II disease achieved 61% survival, compared with only 22% in stage III–IV, underlining the importance of early detection. Tumor location did not significantly affect survival, in agreement with other international studies [26,27]. Although many international studies report survival differences between colon and rectal cancer, no such difference was observed in our cohort. This may be partly explained by the relatively small sample size and the predominance of advanced-stage presentations, which likely overshadowed potential location-related effects. The apparent discrepancy between Chi-square and Kaplan–Meier results likely reflects methodological differences, since Chi-square tests distributional differences in grouped survival data, whereas Kaplan–Meier assesses time-to-event data and showed no prognostic impact of tumor location.

Nodal involvement was another major determinant of prognosis: 85% of patients had N1–N2 disease, consistent with the predominance of stage III and with inferior survival in node-positive subgroups. This mirrors global evidence that lymph-node metastasis markedly reduces survival [28,29]. Age also influenced outcomes, with patients >70 years showing worse survival, reflecting comorbidities and lower tolerance for adjuvant therapies. Similar stage-shifts and treatment disparities in elderly cohorts are well documented in population-based studies [27]. Because overall survival was defined as death from any cause, non-cancer-related mortality likely contributed to the poor outcomes observed in older patients. This is consistent with the high prevalence of cardiovascular and other comorbidities in this population, which may have influenced survival independently of tumor characteristics. Cardiovascular comorbidities and vascular complications may further contribute to poor outcomes, underscoring the value of perioperative risk assessment [30,31].

Sex did not significantly affect prognosis, although men showed a slightly steeper decline after 72 months. While some registries have suggested modest female survival advantages, particularly in younger cohorts, such differences appear to diminish in more recent analyses [22,26]. Histologic grade correlated with outcome: well-differentiated tumors had the best prognosis, while poorly differentiated tumors had the worst. Histologic subtype was not independently predictive, consistent with evidence that poor differentiation and mucinous features are adverse mainly due to association with advanced stage [24]. Recent reports of aggressive behavior in well-differentiated gastrointestinal neuroendocrine tumors highlight the limitations of histologic grading alone [32].

Although margin status (R0 vs. R1/R2) and circumferential resection margin were not systematically recorded, complete R0 resection with adequate nodal harvest remains the strongest surgical predictor of long-term survival in non-metastatic CRC [9,20]. Our previous surgical reports on mesenteric cysts and complex appendiceal conditions [33,34] similarly emphasized the variability of abdominal oncology and the importance of multidisciplinary strategies [35,36].

Multivariate analysis confirmed advanced stage, emergency surgery, and incomplete resection as independent predictors of poor survival, while age >70 years was associated with increased mortality and prolonged hospitalization. In contrast, sex, tumor site, and residence had no independent prognostic impact.

Overall, our findings align with SEER and EUROCARE data, confirming stage, nodal status, age, grade, and resection status as the dominant prognostic factors in CRC. The lower survival observed in this Romanian cohort compared with Western Europe and the USA likely reflects late presentation, limited access to multimodal adjuvant therapy, and systemic barriers in oncology care [3,26].

#### Quality of Surgery and Pathology Considerations

In addition to systemic and stage-related factors, the unexpectedly low 5-year overall survival of 61% among stage I–II patients may also reflect limitations in surgical and pathological quality assurance. During the study period, key indicators such as circumferential resection margin (CRM) status in rectal cancer and the total number of lymph nodes harvested were not systematically documented. Inadequate margin assessment or insufficient nodal evaluation can lead to understaging, incomplete resections, and suboptimal selection for adjuvant therapy. These inconsistencies, characteristic of the Romanian surgical and pathological context in 2003–2005, may partly explain the poorer-than-expected survival outcomes in early-stage disease. Standardized reporting and adherence to modern oncological quality indicators, as emphasized in NCCN and ESMO guidelines, are now considered essential to optimize survival.

### 4.6. Screening Context

At the time of this study (2003–2005), Romania lacked an organized colorectal cancer (CRC) screening program, with population-based initiatives introduced only in recent years. Consequently, most patients in our cohort presented with advanced-stage disease, in line with registry data showing higher mortality and lower survival in Eastern compared with Western Europe [6,37,38]. By contrast, the United States and most EU countries had already implemented fecal occult blood testing (FOBT) or fecal immunochemical testing (FIT), followed by colonoscopy for positive results. These strategies have consistently reduced both incidence and mortality by enabling detection of adenomas and early-stage cancers [3,39,40]. SEER data illustrate a steady decline in CRC mortality in the U.S., largely driven by screening uptake and improved treatment [6,40], while EU countries with long-standing, high-coverage programs report clear survival advantages [3,38].

The absence of organized screening in Romania during the study period likely contributed to late-stage diagnosis and the low survival rates observed in our series. Opportunistic screening, when available, was sporadic, hospital-based, and largely limited to urban populations. From a public health standpoint, these findings highlight the urgent need for nationwide, FIT-based CRC screening with adequate colonoscopy capacity, as recommended by the European Commission [3]. Clinically, systematic screening would allow earlier diagnosis, a higher proportion of R0 resections, and improved alignment of surgical and oncological care with international standards, ultimately increasing survival.

### 4.7. Regional Disparities and the Molecular Era

Our data were derived from a university hospital setting, where access to surgical expertise and multidisciplinary care was relatively advanced for the early 2000s. This context may not fully reflect conditions in smaller regional or district hospitals, where limited infrastructure, reduced availability of specialized personnel, and restricted access to modern therapies have long contributed to delayed diagnosis and poorer outcomes. Such disparities likely explain part of the variation in stage distribution and survival across Romania. Patients from rural or underserved areas often face delayed access to colonoscopy, pathology services, and adjuvant therapy, in contrast with larger centers where guideline-based multimodality care is more feasible. These inequities mirror broader European patterns, with EUROCARE and CONCORD studies documenting persistent survival gaps between Western and Eastern Europe [37,41].

Another limitation of our cohort is the absence of molecular data, as KRAS, NRAS, BRAF, and microsatellite instability (MSI) testing were not available in Romania during 2003–2005. These biomarkers are now recognized as essential for prognosis and treatment guidance: RAS mutations predict resistance to anti-EGFR therapies, BRAF V600E mutations confer poor prognosis but are targetable, and MSI-high/dMMR tumors not only carry favorable outcomes in localized disease but also represent candidates for immunotherapy in advanced stages [9,20,42,43]. The lack of molecular profiling restricts our ability to stratify outcomes beyond conventional clinicopathologic parameters. Future Romanian studies should therefore incorporate routine biomarker testing to align with NCCN, ESMO, and ASCO recommendations, enabling precision oncology and ensuring comparability with international datasets.

### 4.8. Public Health Implications

Our findings confirm the substantial burden of colorectal cancer in Romania, with high mortality-to-incidence ratios and a predominance of late-stage diagnoses reflecting insufficient early detection. Strengthening nationwide screening—preferably FIT-based with colonoscopy follow-up—together with public awareness campaigns should be considered a public health priority.

Equally important is the alignment of national strategies with European initiatives such as *Europe’s Beating Cancer Plan* and the *Mission on Cancer* [44]. These frameworks promote harmonized approaches to prevention, early detection, and treatment across the EU, aiming to reduce inequalities and improve survival. Romania’s integration into these programs could expand access to organized screening, modern therapies, and multidisciplinary care, thereby narrowing current survival gaps.

### 4.9. Future Research

Future studies should focus on establishing multicenter, nationwide registries to comprehensively capture incidence, treatment patterns, and outcomes. Such platforms would allow real-time monitoring of trends, benchmarking against European and international standards, and provide robust evidence for health policy.

Integration of epidemiological, clinical, and molecular data into national databases is also crucial. Incorporating biomarker testing (e.g., KRAS, NRAS, BRAF, MSI) would align Romanian oncology with international practice and accelerate the adoption of precision medicine. In addition, systematic evaluation of pilot CRC screening programs introduced after 2019 should be prioritized, assessing participation rates, early-stage detection, and downstream effects on survival to guide the design and implementation of effective nationwide screening.

### 4.10. Historical Context

The study period (2003–2005) predates the introduction of structured CRC screening in Romania and the widespread adoption of minimally invasive surgery, as well as the availability of oxaliplatin-based chemotherapy and molecular testing. As a result, most cases were diagnosed at advanced stages and treated with open procedures. By contrast, the current landscape is markedly different: robotic and laparoscopic techniques are increasingly used, and systemic therapy has expanded to include targeted agents and immunotherapies.

While these findings no longer mirror current clinical practice, their value lies in providing a historical benchmark that illustrates how clinical outcomes have evolved over time. Such data can serve as a reference point for contemporary cohorts and health system development, rather than as guidance for modern patient care. Our cohort therefore provides valuable baseline data, enabling future comparisons and highlighting the magnitude of progress achieved in surgical oncology and systemic therapies over the past two decades.

Colorectal cancer in Romania remains characterized by late-stage presentation and lower survival rates compared with Western Europe. Radical resection and early-stage diagnosis emerged as the strongest predictors of favorable long-term outcomes. The absence of minimally invasive techniques and the limited availability of systemic therapy during the early 2000s likely contributed to higher morbidity and poorer survival rates. Expanding nationwide screening programs and improving access to modern multimodal treatments are essential to close the survival gap. Historical cohorts such as this provide a valuable benchmark for evaluating progress and identifying priority areas for healthcare system improvement.

#### SWOT Analysis (Summary)

**Strengths:**

This study provides one of the most detailed single-center analyses of colorectal cancer in Romania during the early 2000s, with complete long-term follow-up and comprehensive documentation of demographic, clinicopathological, surgical, and survival data in 302 patients. Direct comparison with national and international datasets (Table 12) offers a valuable historical benchmark for understanding the evolution of colorectal cancer management. The robust methodological approach, including multivariate analyses, enhances the reliability of prognostic findings and allows contextualization within broader European trends.

**Weaknesses:**

The single-center, retrospective design limits generalizability and reflects the heterogeneity of colorectal cancer care in Romania at that time. Standardized oncological quality indicators, such as CRM status and lymph node harvest, were incompletely reported. Minimally invasive surgery was not available, and chemotherapy regimens were limited to 5-FU–based protocols with restricted access to oxaliplatin or targeted agents. Molecular profiling was absent, and data on treatment at recurrence were incomplete. These factors contributed to lower survival rates compared with contemporary Western cohorts.

**Opportunities:**

Expanding nationwide screening programs and increasing access to modern multimodal therapies—including minimally invasive surgery, total neoadjuvant therapy, and biomarker-driven systemic treatment—represent clear avenues for improving outcomes. The development of national registries and multicenter collaborations could enhance data quality, allow real-time monitoring of disease trends, and support evidence-based health policy planning. Integration of molecular testing (RAS, BRAF, MSI) would align Romanian oncology with current NCCN and ESMO guidelines.

**Threats:**

Persistent disparities in healthcare infrastructure, delayed implementation of screening, and unequal access to advanced surgical and oncological treatments risk maintaining the survival gap between Romania and Western Europe. Regional inequalities, workforce limitations, and inadequate integration of modern standards of care could hinder progress if not addressed systematically.

## 5. Conclusions

This study provides one of the most detailed single-center analyses of colorectal cancer in Romania during the early 2000s. Our findings confirm the predominance of late-stage diagnosis, the limited feasibility of complete (R0) resections, and the substantial impact of stage, nodal involvement, age, and resection status on survival. With an overall 5-year survival of 38%, markedly lower than in Western Europe or the USA, this cohort highlights systemic barriers to early detection and modern multimodal treatment.

From a clinical perspective, the results emphasize the urgent need for nationwide implementation of colorectal cancer screening programs and wider access to guideline-based therapies, including modern chemotherapy, radiotherapy, and minimally invasive surgical techniques. At the same time, integrating molecular profiling into routine practice will be essential to align Romanian oncology with international standards and to advance precision medicine.

In summary, this study contributes valuable historical data and underlines both the progress achieved in the past two decades and the challenges that remain in bridging survival gaps between Romania and high-income countries.

## Figures and Tables

**Table 1:** Operational definitions applied in the study.

Term	Definition
**Radical resection**	Resection with curative intent aiming for microscopically negative margins (R0).
**Palliative procedure**	Colostomy or limited resections performed without curative intent.
**Emergency surgery**	Surgery performed for acute complications (bowel obstruction, perforation, hemorrhage).
**Survival**	Time from date of surgery to death (any cause) or last follow-up.

**Table 2:** Baseline characteristics of the study cohort (2003–2005).

Variable	*n* (%)/Mean ± SD
**Total patients**	302
**Age, mean (range)**	65 (33–91)
**Age group**	<60: 28%/60–79: 61%/≥80: 11%
**Sex (M/F)**	177/125 (1.43:1)
**Residence (urban/rural)**	206/96 (68%/32%)
**Tumor location**	Colon 62%/Rectum 38%
**Stage at diagnosis**	I–II: 40%/III–IV: 60%

**Table 3:** Survival outcomes by stage and resection type.

Variable	1-Year Survival	3-Year Survival	5-Year Survival
**Overall cohort**	76%	52%	38%
**Stage I–II**	91%	74%	61%
**Stage III–IV**	63%	36%	22%
**R0 resection**	88%	67%	45%
**R1–R2 resection**	59%	28%	18%

**Table 4:** Chi-square analysis of clinicopathological variables and outcomes by tumor location.

**Comparison**	**χ^2^ Value**	**df**	***p*** **-Value**	**Significance**	**Interpretation**
**Location × Emergency vs. Elective surgery**	1.015	2	0.602	n.s.	No association
**Location × Differentiation grade (G1–G3)**	2.998	4	0.558	n.s.	No association
**Location × Stage (I–IV)**	11.385	6	0.077	n.s. (trend)	Borderline, not significant
**Location × Complications (yes/no)**	2.631	2	0.268	n.s.	No association
**Location × Macroscopic form (infiltrative/ulcerated/vegetant)**	6.650	4	0.156	n.s.	No association
**Location × Survival duration (years)**	28.697	16	0.026	**Significant**	Longer-term survival differed by location
**Location × Overall survival (<5 vs. ≥5 years)**	1.055	2	0.590	n.s.	No association

Legend: df = degrees of freedom; n.s. = not significant.

**Table 5:** Logistic regression analysis of prognostic factors for 5-year survival.

Factor	*p*-Value	Significance/Interpretation
**Age > 70 years**	0.025	Associated with poor prognosis
**Male sex**	0.880	NS
**Rural residence**	0.470	NS
**Stage III–IV**	<0.001	Strong predictor
**Emergency surgery**	<0.001	Strong predictor
**R1–R2 resection**	0.002	Associated with poor prognosis
**Rectal location**	0.700	NS

Legend: NS = not significant.

**Table 6:** Cox regression analysis of prognostic factors for overall survival.

Factor	*p*-Value	Significance/Interpretation
**Age > 70 years**	0.020	Associated with higher mortality risk
**Male sex**	0.630	NS
**Rural residence**	0.320	NS
**Stage III–IV**	<0.001	Strong predictor of poor survival
**Emergency surgery**	<0.001	Strong predictor of poor survival
**R1–R2 resection**	0.004	Associated with poor prognosis
**Rectal location**	0.820	NS

Legend: NS = not significant.

**Table 7:** Ordinal logistic regression analysis of predictors for advanced stage at diagnosis.

Factor	*p*-Value	Significance/Interpretation
**Age > 70 years**	0.010	Older patients more often diagnosed at late stage
**Male sex**	0.820	NS
**Rural residence**	0.040	Higher risk of late stage
**Rectal location**	0.850	NS
**Emergency surgery**	0.001	Strong predictor of advanced stage

Legend: NS = not significant.

**Table 8:** Multinomial logistic regression analysis of predictors for surgical strategy.

Factor	Surgical Strategy (vs. Left Hemicolectomy)	*p*-Value	Interpretation
**Tumor in right colon**	Right hemicolectomy	<0.001	Strong predictor
**Tumor in rectum**	Abdominoperineal resection	<0.001	Strong predictor
**Stage III–IV**	Palliative procedure	0.002	Advanced stage linked to palliation
**Age > 70 years**	Palliative procedure	0.090	NS (trend)
**Complicated tumor**	Emergency surgery (any type)	0.004	Significant

Legend: NS = not significant.

**Table 9:** Poisson regression analysis of predictors for postoperative complications.

Factor	*p*-Value	Significance/Interpretation
**Age > 70 years**	0.040	More complications
**Male sex**	0.620	NS
**Stage III–IV**	0.010	Higher risk
**Emergency surgery**	<0.001	Strong predictor
**R1–R2 resection**	0.240	NS
**Rectal location**	0.800	NS

Legend: NS = not significant.

**Table 10:** Negative binomial regression analysis of predictors for length of hospital stay.

Factor	*p*-Value	Significance/Interpretation
**Age > 70 years**	0.020	Longer hospitalization
**Male sex**	0.740	NS
**Stage III–IV**	0.001	Longer hospitalization
**Emergency surgery**	<0.001	Strong predictor
**Complications (yes)**	<0.001	Very strong predictor
**R1–R2 resection**	0.220	NS

Legend: NS = not significant.

**Table 11:** Logistic regression analysis of predictors for complete resection (R0).

Factor	*p*-Value	Significance/Interpretation
**Stage III–IV**	<0.001	Lower chance of R0
**Emergency surgery**	0.010	Poor predictor of complete resection
**Rectal location**	0.650	NS
**Age > 70 years**	0.880	NS

Legend: NS = not significant.

**Table 12:** Comparison between our historical results (Romania 2003–2005) and recent international data for colorectal cancer.

Indicator	Romania (2003–2005, Our Study)	Romania (2022, OECD/EC 2025 Report) [2,3]	EU-27 (2020–2022, JRC/ECIS) [4,7]	USA (SEER 2018–2020) [5]	Observation
Incidence (per 100,000)	~7–8	~33–34 (≈11,700 new cases)	30–45 (variation)	~35–40	Major increase in RO
Mortality (per 100,000)	~5–5.5	~23 (≈7900 deaths)	Declining trend	~13–15	RO much higher
Mortality/Incidence ratio (M/I)	≈0.67	≈0.70	~0.40	~0.35	Late diagnosis in RO
Male/Female ratio	1.43:1	≈1.4:1	≈1.4:1	≈1.4:1	Consistent
Mean age at diagnosis	65 years	>65 years	similar	similar	Concordant
Trend	Increasing	Increasing	Declining	Declining	Divergence RO vs. West

## Data Availability

The data presented in this study are available on request from the corresponding author. The data are not publicly available due to patient confidentiality.
